# Monocrystalline silicon solar cell efficiency dependence on potassium silicate additive in texturing process with a study on the effect of the active material

**DOI:** 10.1371/journal.pone.0348411

**Published:** 2026-05-15

**Authors:** Salwa Abdel Naser, Ahmed A. Zaki Diab, Saleh Al Dawsari, Mohamed A. Ismeil, Mohamed Zahran

**Affiliations:** 1 Electrical Engineering Department, Faculty of Engineering, Minia University, Minia, Egypt; 2 The joint National Egyptian-Chinese Renewable Energy Laboratory, Sohag, Egypt; 3 Department of Mechatronics Engineering, Faculty of Engineering, Nahda University in Beni Suef, Beni Suef, Egypt; 4 School of Engineering, Cardiff University, Cardiff, United Kingdom; 5 Electrical Engineering Department, College of Engineering, Najran University, Najran, Saudi Arabia; 6 Electrical Engineering Department, Faculty of Engineering, King Khalid University, Abha, Saudi Arabia; 7 Ministry of Higher Education and Scientific Research, Cairo, Egypt; National Research Centre, EGYPT

## Abstract

Texturing is a critical process for enhancing monocrystalline silicon solar cell efficiency by reducing surface reflectance and improving light absorption. This study investigates the effect of introducing potassium silicate (K₂SiO₃) as an additive during alkaline texturing while simultaneously reducing the concentration of isopropyl alcohol (IPA)—a volatile organic solvent commonly employed to control anisotropic etching, ensuring uniform pyramid formation. The analysis examines surface morphology, particularly the pyramid structures formed on silicon wafers, and evaluates changes in photovoltaic performance with and without K₂SiO₃ addition. Wafers were weighed before and after texturing to quantify material removal. Weight loss measurements showed that texturing with 500 mL IPA alone (without K₂SiO₃) resulted in excessive etching (1.057 g, exceeding the optimal range of 0.6–1.0 g), while addition of 5 g K₂SiO₃ with reduced IPA (250 mL) yielded controlled weight loss (0.942 g) within the acceptable range. This demonstrates that potassium silicate regulates the etching process, preventing over-texturing and improving surface quality. Following plasma-enhanced chemical vapor deposition (PECVD), the thickness and refractive index of the deposited SiNₓ anti-reflection coating were measured using ellipsometry, yielding values of 87.1 nm and 1.99 for wafers textured without K₂SiO₃ (500 mL IPA), and 81.6 nm and 1.93 for those processed with K₂SiO₃ (250 mL IPA + 5 g K₂SiO₃). Completed solar cells were characterized using current–voltage (I–V) measurements to determine electrical parameters and efficiency. All experiments were conducted at the Egyptian-Chinese Laboratory for Renewable Energy Applications. Pyramid homogeneity was further analyzed using scanning electron microscopy (SEM). Statistical analysis including ANOVA and Tukey HSD post-hoc testing was performed to validate the significance of observed efficiency improvements.

## 1 Introduction

The escalating global energy demand, coupled with the environmental consequences of fossil fuel consumption, has necessitated an accelerated transition toward renewable energy sources [1,2]. Many nations are confronting energy shortages, and with continued population growth, electricity demand threatens to outstrip local supply—an imbalance that underscores the urgency of adopting sustainable alternatives [3,4]. Among renewable options, solar photovoltaic (PV) technology has gained particular prominence, especially in Arab countries endowed with abundant solar irradiation throughout the year [5,6]. Globally, solar PV has emerged as the third most significant renewable energy source for electricity generation after hydropower and wind power [7]. The IPCC report highlights that solar and wind energy costs decreased by up to 85% between 2010–2019, making them increasingly competitive. It emphasizes the urgent need to peak global emissions by 2025 and reduce them 43% by 2030 to limit warming to 1.5°C [8]. In 2024, global renewable power capacity grew by a record 585 GW (15.1% annual growth), with solar alone contributing 451.9 GW. Renewables, led by solar and wind, accounting for over 90% of all new power capacity added worldwide [9].

Monocrystalline silicon solar cells dominate the PV market due to their mature fabrication processes, established technology infrastructure, and continually improving efficiency metrics [10]. Consequently, substantial research efforts have focused on optimizing manufacturing processes to achieve higher efficiencies at lower cost [11,12]. Silicon, with its diverse structural morphologies, remains the material of choice for electronic devices and photovoltaic applications [13].

A primary loss mechanism in silicon solar cells is optical reflection at the front surface, which can account for a significant portion of incident energy loss [14,15]. Two complementary approaches address this issue: anti-reflective coatings (ARC) and surface texturing [1620,21]. For instance, Khalil and Mohamed [16] explored a cost-effective sol-gel method to deposit porous silica anti-reflective coatings on glass protectors for PV modules. Beyond optical losses, thermal management presents additional challenges; elevated operating temperatures degrade cell performance, as modeled by Hamdy et al. [17] and experimentally demonstrated by Elsheikh et al. [18]. PV industries therefore employ advanced metallization products to minimize electrical losses [19] alongside ARC and surface texturing to reduce optical losses and enhance overall efficiency [20,21]. Surface texturing has been accomplished through various techniques [22]. In industrial practice, texturing serves multiple functions including surface cleaning [23] and removal of saw damage incurred during wafer slicing [24], typically achieved through wet chemical treatment.

### 1.1 Purpose and mechanisms of texturing

**Fig 1:**
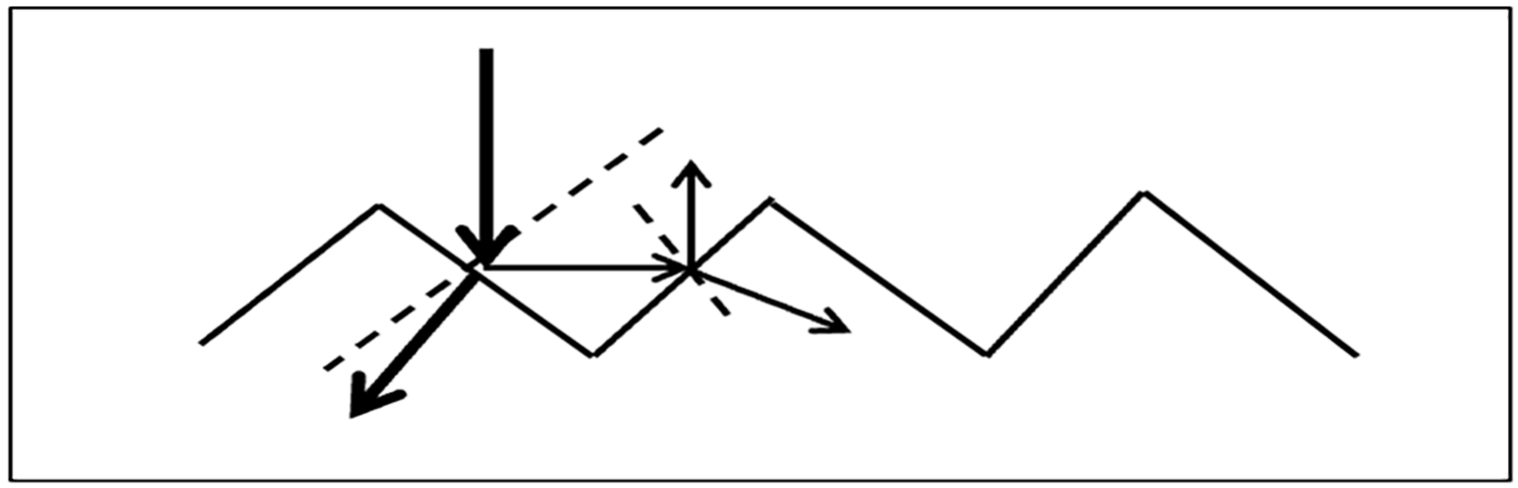
Schematic illustration of enhanced photon path length on a textured monocrystalline silicon surface, demonstrating improved light trapping after the texturing process.

Texturing serves two primary functions in monocrystalline silicon solar cells: (1) reducing surface reflection by creating microscopic pyramidal structures that trap incident light, and (2) enhancing internal light absorption by increasing the effective optical path length through multiple internal reflections (Fig 1). Together, these effects significantly improve photo-response and overall conversion efficiency. As a result, random texturing has become a standard technology for enhancing conversion efficiency in monocrystalline silicon solar cells by increasing light absorption [25,26].

### 1.2 Advances in texturing additives

Texturing, the first process in industrial solar cell fabrication, reduces reflection losses [27] and has been extensively studied and optimized using various chemical additives. Basu et al. [28] demonstrated that utilizing a small amount of liquid potassium silicate as an additive to the conventional potassium hydroxide (KOH) and isopropyl alcohol (IPA) alkaline texturing process achieves optimized texture with low solar weighted average reflectance (WAR) of 11.4% to 12.2%, which is further reduced to approximately 2% after application of a standard anti-reflection coating. Industrial-grade solar cells fabricated using this advanced texturing method achieved high conversion efficiencies exceeding 18% [28]. Anisotropic etching of silicon has gained widespread acceptance in the manufacture of industrial monocrystalline silicon solar cells to decrease reflection losses from the front surface. Many studies have investigated alternative texturing additives to improve process efficiency and reduce costs. Birmann et al. [29] introduced a novel methodology for texturing by combining KOH with 1,4-cyclohexanedione (CHX). Mayer et al. [30] explored different approaches employing polymer concentrates and aromatic compounds in KOH solutions for texturing crystalline silicon. Quiebras et al. [31] achieved a cell efficiency of 17.6% using a mixture of KOH and high boiling alcohol (HBA). Lee et al. [32] focused on developing a simplified, high-efficiency, and low-cost fabrication methodology for industrial crystalline silicon solar cell production. Their work described specific modifications to standard industrial processes for texturing, diffusion, and contact formation. An optimized surface texturing process achieved high-quality surface morphology with minimal etching depth of approximately 5.5 μm per side, with an optimized etching rate of 0.37 μm/min. The collectively implemented optimizations yielded a solar cell with a final conversion efficiency of 17.1% [32]. A significant challenge in conventional texturing is the high consumption of IPA due to evaporation, as the process typically operates at 85 °C—above the boiling point of IPA. Many researchers have worked to reduce the amount of added IPA [33]. Han et al. [34] presented a comparative evaluation of various wet texturing methods using alkaline solutions with and without additives, reporting that KOH-based texturing with additives achieves comparable pyramid formation in only 15 minutes, compared to 30 minutes for conventional NaOH + IPA processes.

### 1.3 Present study and objectives

The study was conducted at the Egyptian-Chinese National Renewable Energy Laboratory in Sohag, Egypt, which aims to achieve high-efficiency silicon solar cells while reducing production costs through continuous development of manufacturing processes [27]. The objective is to obtain improved manufacturing efficiency for the resulting solar cells while reducing production cost through the introduction of new chemical materials whose effects are being systematically evaluated. Preliminary results indicate that the use of potassium silicate in the texturing process increased the efficiency of solar cells produced by the Egyptian-Chinese laboratory. The efficiency achieved with the conventional method (500 mL IPA, no silicate) was 17.17%, while the addition of potassium silicate (250 mL IPA + 10 g K₂SiO₃) increased efficiency to 18.18%. This study provides comprehensive statistical analysis and quantitative morphological characterization to validate these improvements and guide process optimization for industrial implementation.

## 2. Materials and methods

### 2.1 Experimental design and measurement techniques

This study investigates the effect of potassium silicate (K₂SiO₃) as a co-additive in the alkaline texturing process for monocrystalline silicon solar cells, with the objective of reducing isopropyl alcohol (IPA) consumption while maintaining or improving cell efficiency. Four experimental conditions were evaluated: (1) without active material (control), (2) with 500 mL IPA only, (3) with 250 mL IPA + 5 g K₂SiO₃, and (4) with 250 mL IPA + 10 g K₂SiO₃. This study does not involve human or animal subjects and therefore ethical approval was not required.

To assess the impact of these conditions on wafer morphology and device performance, the following measurements were performed:

- Mass loss: Wafers were weighed before and after texturing using a balance (Fig 5) to quantify material removal. Acceptable weight loss for the texturing process ranges from 0.6 to 1.0 g.
- Surface morphology: Pyramid distribution and homogeneity were examined using optical microscopy and further analyzed by scanning electron microscopy (SEM).
- Sheet resistance: Measured using a four- probe system (Fig 7).
- Anti-reflective coating properties: Thickness and refractive index of the deposited silicon nitride (Sinnₓ) layer were determined using an ellipsometer (Fig 11).
- Electrical characterization: Completed solar cells were tested using a current–voltage (I–V) measurement system (Fig 13) under standard test conditions (25 °C, AM 1.5, 1000 W/m^2^) to determine efficiency, fill factor, series resistance, shunt resistance, and maximum power output.

### 2.2 Materials and wafer specifications

The chemicals used in the texturing process included isopropyl alcohol (IPA as active material), potassium hydroxide (KOH), potassium silicate (K₂SiO₃), hydrochloric acid (HCl, 3.88%), and hydrofluoric acid (HF, 4.44%). All chemicals were of semiconductor-grade purity.P-type monocrystalline silicon wafers doped with gallium were used as the substrate. Wafer specifications are provided in Table 1.

**Table 1:** The wafer characterization.

Parameter	Value
Area (mm^2^)	156.75*156.75
Thickness (μm)	180 + 20/-10
Type & dope	P- Gallium
Resistivity (Ω. cm)	0.5-1.5

### 2.3 Solar cell fabrication process

**Fig 2:**
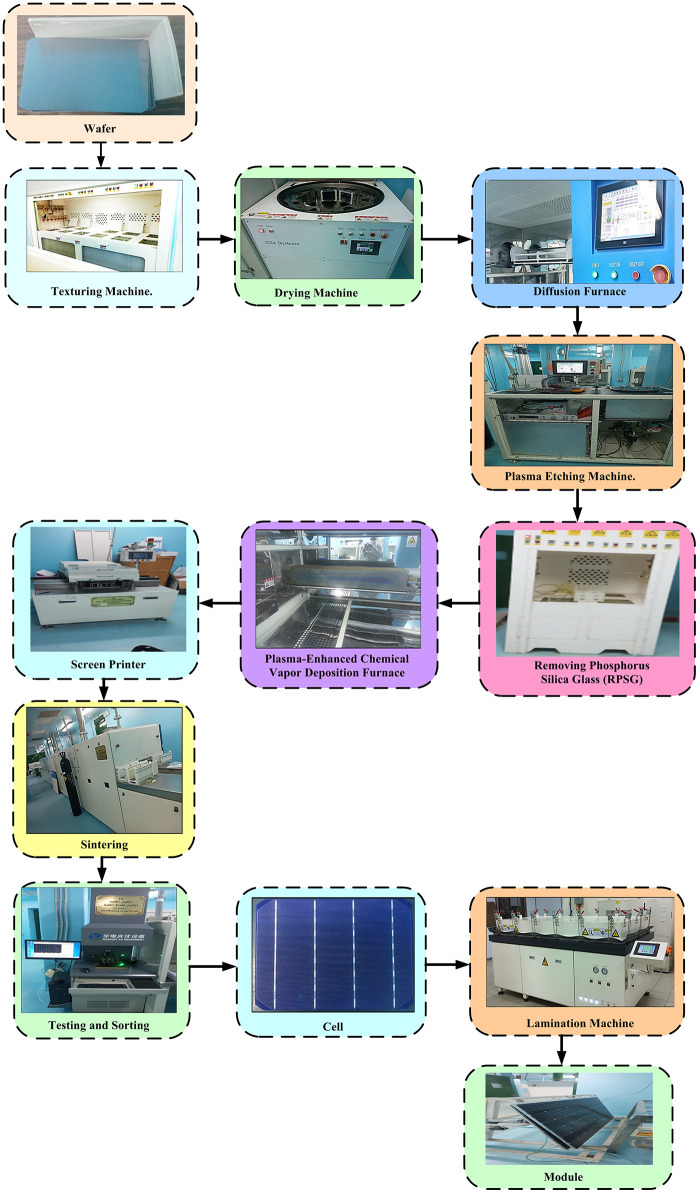
Complete process flow for solar cell fabrication at the Egyptian-Chinese Laboratory.

All fabrication steps were conducted at the Egyptian-Chinese National Renewable Energy Laboratory in Sohag, Egypt, within a Class 1000 clean room environment to minimize contamination. Fig 2 illustrates the complete process flow for crystalline silicon solar cell fabrication as implemented in the laboratory, from incoming wafer inspection to final module assembly.

#### 2.3.1 Texturing process.

**Fig 3:**
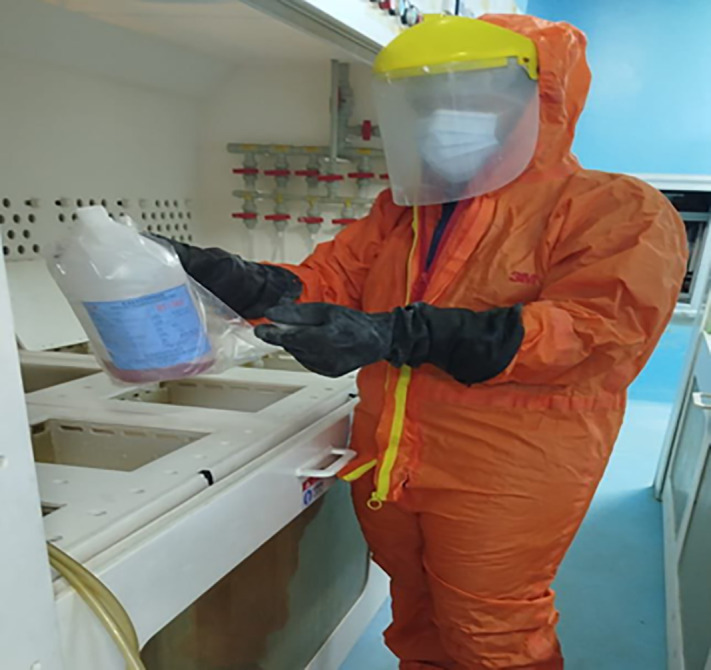
Clean room safety attire required for laboratory access.. Clean room safety attire required for laboratory access.

The experiment was conducted inside the clean room, which was designed to minimize contamination of the process and materials. This is achieved by removing or reducing sources of contamination, for example (particulate, chemicals, biological and radiation). All safety precautions were taken within the venue, and strict adherence to wearing the designated uniform for the experiment (gas mask, protective mask, acid and alkaline resistance overalls and acid and alkaline resistance gloves), as shown in Fig 3.

**Fig 4:**
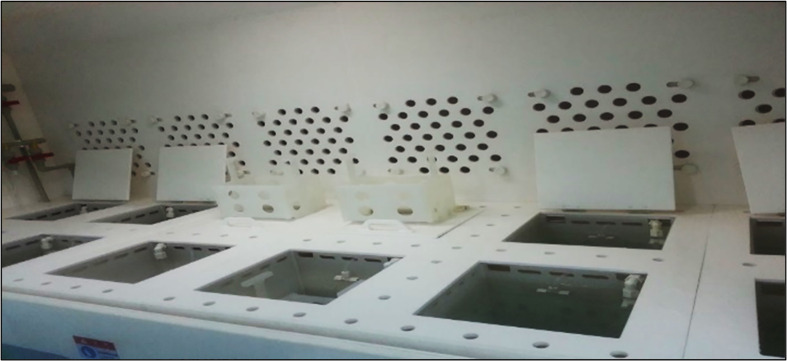
Texturing process tanks for wet chemical etching.

Texturing was performed using the wet chemical etching system shown in Fig 4, consisting of eight tanks (28 × 28 cm cross-section, 32 cm depth).

The process sequence was as follows:

a) A 25 L tank was filled with deionized water and heated to 85 °C. Potassium hydroxide (KOH, 0.1678 mol) was added, and the solution was stirred until homogeneous. Three silicon wafers were placed in the tank for 20 min to activate the solution. Subsequently, all wafers were cleaned to remove saw damage by immersion in the tank for 4 min.
b) The amount of KOH was doubled to a total of 0.3357 mol. After thorough mixing, a cassette containing the wafers was placed inside the tank.
c) Isopropyl alcohol (IPA, 500 mL) was added as the active material, and a second cassette was processed for 10 min.
d) Steps (a) and (b) were repeated in a separate tank, but with the active material (IPA) reduced to 250 mL and 5 g of potassium silicate (K₂SiO₃) added. A third cassette was then processed for 10 min.
e) An additional 5 g of potassium silicate was added to the tank, bringing the total amount to 10 g. A fourth cassette was processed for 10 min at 85 °C.
f) All silicon wafers were immersed in 4.44% hydrofluoric acid (HF) for 4 min at 25 °C to remove oxide layers.
g) All silicon wafers were immersed in 3.88% hydrochloric acid (HCl) for 4 min at 25 °C to remove organic residues.

**Fig 5:**
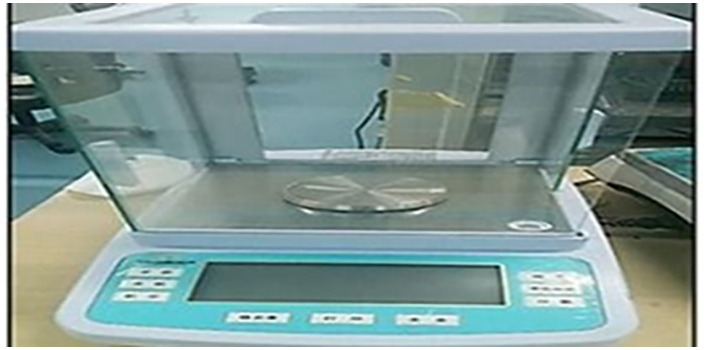
Sensitive balance used for wafer mass measurement before and after texturing.

Notes should be considered regarding the cleaning and drying procedures during wafer preparation:

- After each processing step, the silicon wafers are thoroughly rinsed with deionized water and flushed with nitrogen for 3 minutes to remove residual chemicals and prevent contamination.
- Once the texturing process was completed, the wafers were dried using a drying machine for 5 minutes at 51 °C under a nitrogen atmosphere. The use of nitrogen accelerates the drying process and reduces the risk of oxidation.
- After drying, A sensitive balance, shown in Fig 5, is used to weigh the wafers before and after the texturing process. The weight loss after texturing should be in the range of (0.6–1) g.

#### 2.3.2 Diffusion process.

**Fig 6:**
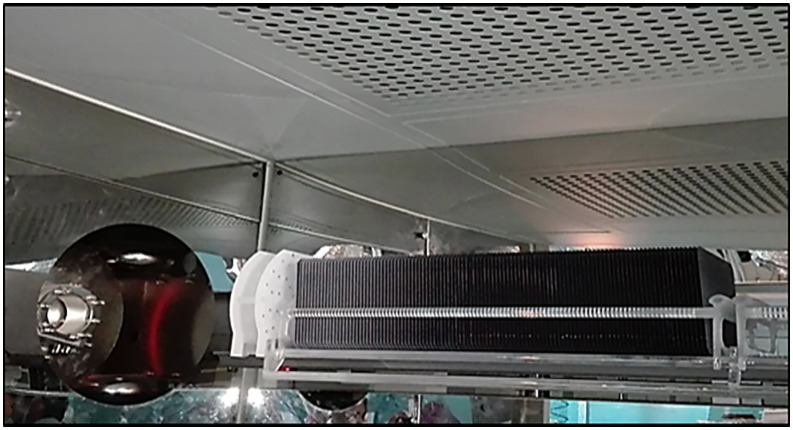
Diffusion furnace for p-n junction formation.

Following texturing, the p-n junction was formed by phosphorus diffusion using the diffusion furnace shown in Fig 6.

**Fig 7:**
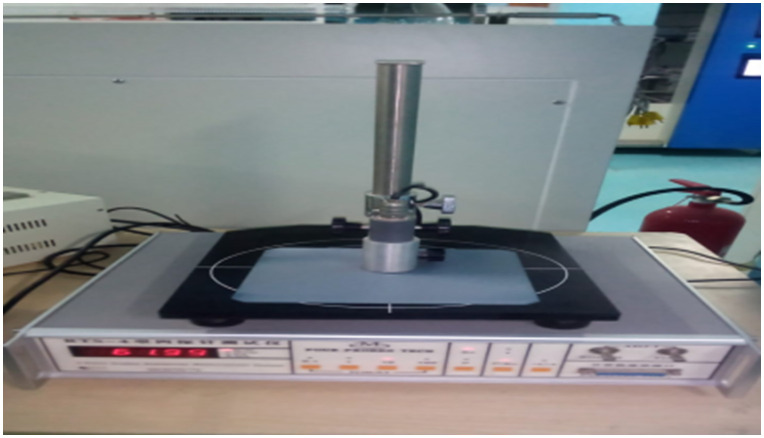
Four-point probe tester for sheet resistance measurement.

The furnace is divided into five independently controlled temperature zones. After diffusion, sheet resistance was measured using a four-point probe tester (Fig 7).

#### 2.3.3 Plasma edge etching.

**Fig 8:**
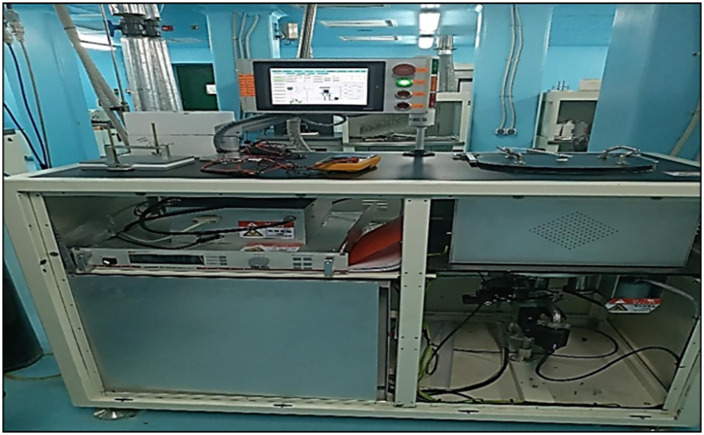
Plasma etching machine for edge isolation.

The phosphorus-doped layer deposited on wafer edges during diffusion was removed using a plasma etching machine (Fig 8) to prevent short-circuiting of the cell.

#### 2.3.4 Removal of phosphosilicate glass (RPSG).

**Fig 9:**
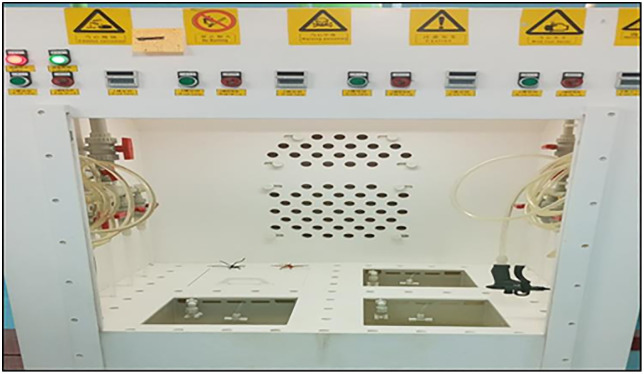
Wet chemical tanks for RPSG removal.

The phosphosilicate glass layer formed during diffusion was removed using the wet chemical system shown in Fig 9. This step is critical for subsequent anti-reflective coating and metallization processes.

#### 2.3.5 Plasma-enhanced chemical vapor deposition (PECVD) furnace.

**Fig 10:**
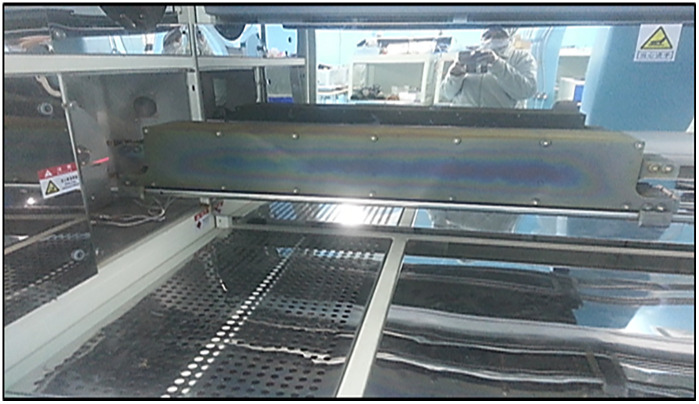
PECVD furnace for SiNₓ deposition.

Anti-reflective coating is important for solar cell performance as it provides a high photo-current by minimizing reflectance. Fig 10 shows the (PECVD) furnace.

**Fig 11:**
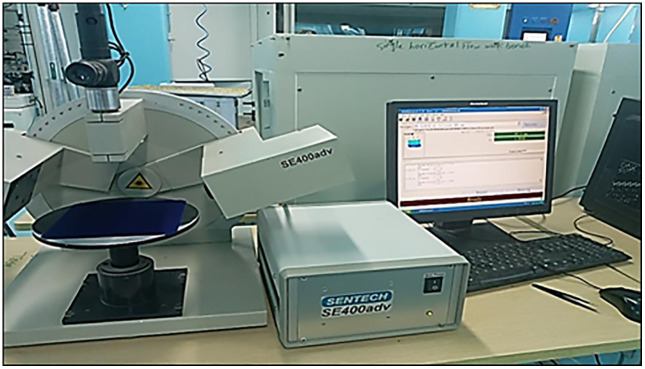
Ellipsometer for SiNₓ layer characterization.

Following deposition, wafer color was inspected (target: uniform blue), and coating thickness and refractive index were measured using an ellipsometer (Fig 11).

#### 2.3.6 Metallization *by* screen printing.

**Fig 12:**
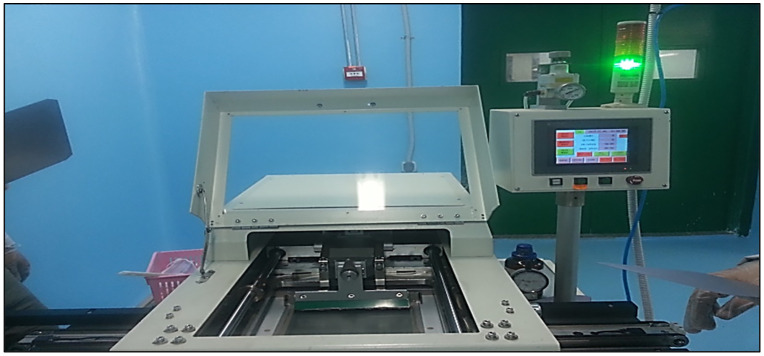
Screen printing machine.

Front and rear contacts were applied using a three-stage screen printing process (Fig 12). The function of each printer is detailed in Table 2.

**Table 2:** Function of each screen printer in the metallization sequence.

Wafer Appearance	Description	Printer
As shown in Fig 13.a).	Printing of silver-aluminum paste (80% silver, 20% aluminum) on the backside of the wafer to form the bus bars, which are subsequently used for interconnecting cells via welding.	a)First Screen Printer
As shown in Fig 13.b).	Application of aluminum paste over the entire backside surface of the wafer, excluding the bus bars printed during the first stage. This layer functions as a back surface field (BSF), enhancing electrical performance and improving rear contact quality.	b)Second Screen Printer
As shown in Fig 13.c).	Printing of pure silver paste on the front side of the wafer to form the grid pattern, comprising fingers and bus bars. This front metallization layer is responsible for collecting photo-generated electrons upon exposure to sunlight.	**c)** Third Screen Printer

Table 2 outlines the function of each screen printer used during the metallization process of the solar cell.

Following printing, wafers were passed through a sintering furnace to dry and fire the contacts, ensuring good ohmic contact formation.

#### 2.3.7 Electrical testing.

Completed cells were tested using an I–V cell tester (Fig 14) under standard conditions (25 °C, AM 1.5, 1000 W/m^2^) to determine efficiency, fill factor, series resistance, shunt resistance, and maximum power output.

## 3 Results

A comprehensive study was conducted to investigate the effect of adding small quantities of liquid potassium silicate to the conventional KOH/IPA alkaline texturing process on both the homogeneity of the pyramidal surface texture and the efficiency of the resulting solar cells.The experiment was performed under four conditions: without the addition of IPA, with the addition of 500 mL of IPA, and with the addition of reduced IPA volume (250 mL) supplemented with potassium silicate at two different concentrations (5 g and 10 g). The influence of potassium silicate on the morphology of pyramids formed on the silicon wafer surface was systematically examined.

### 3.1 Texturing process without adding any active material

In the absence of added active material (IPA), the pyramids formed on the silicon wafer surface were completely heterogeneous. This lack of uniformity is attributed to incomplete reaction kinetics and insufficient surface corrosion, with the corrosion rate falling below the permissible limits. Consequently, numerous voids were observed on the wafer surface, indicating poor texturing quality and presaging low efficiency in the resulting solar cells. These observations were corroborated by subsequent measurements and analyses. Fig 15 presents an optical microscopy image of the wafer surface, while Fig 16 shows the corresponding scanning electron microscopy (SEM) image illustrating the homogeneity and size distribution of the pyramids. The electrical performance of the fabricated solar cells is presented in Fig 17. Fig 17(a) shows the screenshot of the Solar Cell Module Tester (SCMT), while Fig 17(b) presents the corresponding MATLAB plot of the current–voltage (I–V) and power–voltage (P–V) characteristics derived from the measured data. Additionally, the complete voltage, current, and power measurement data are provided in S1 Appendix.

### 3.2 Texturing process with add 500 ml of active material (IPA)

Upon the addition of 500 mL of the active material (IPA), the pyramids formed on the silicon wafer surface exhibited somewhat improved uniformity, leading to an increase in the efficiency of the resulting cell. However, the observed corrosion rate on the wafer surface exceeded acceptable limits. Fig 18 presents an optical microscopy image of the wafer surface. Fig 19 shows the corresponding scanning electron microscopy (SEM) image illustrating the homogeneity and size distribution of the pyramids. The electrical performance of the fabricated solar cells is presented in Fig 20. Fig 20(a) shows the screenshot of the Solar Cell Module Tester (SCMT), while Fig 20(b) presents the corresponding MATLAB plot of the current–voltage (I–V) and power–voltage (P–V) characteristics derived from the measured data. Additionally, the complete voltage, current, and power measurement data are provided in S2 Appendix.

### 3.3 Texturing process with 250 mL of isopropyl alcohol (IPA) and 5 g of potassium silicate

In this case, the volume of IPA was reduced to 250 mL, supplemented with 5 g of potassium silicate (K₂SiO₃). The pyramids formed on the silicon wafer surface were exceptionally uniform, with no observable voids. Additionally, an extension of the texturing bath lifetime was noted. The reduction in IPA consumption contributes to lower production costs while simultaneously enhancing the efficiency of the resulting solar cells. The improved homogeneity of the pyramidal surface texture facilitated enhanced internal light absorption. Fig 21 presents an optical microscopy image of the wafer surface. Fig 22 shows the corresponding scanning electron microscopy (SEM) image illustrating the homogeneity and size distribution of the pyramids. The electrical performance of the fabricated solar cells is presented in Fig 23. Fig 23(a) shows the screenshot of the Solar Cell Module Tester (SCMT), while Fig 23(b) presents the corresponding MATLAB plot of the current–voltage (I–V) and power–voltage (P–V) characteristics derived from the measured data. Furthermore, the complete voltage, current, and power measurement data are provided in S3 Appendix.

### 3.4 Texturing process with 250 mL of isopropyl alcohol (IPA) and 10 g of potassium silicate

The addition of potassium silicate at a concentration of 10 g was found to yield a slight enhancement in solar cell efficiency. However, while the marginal improvement observed at 10 g relative to 5 g suggests a potential concentration-dependent benefit, the effect is not statistically significant given the current sample size. The data indicate that the majority of the performance gain is achieved with the addition of 5 g, with further benefits diminishing at higher concentrations. Fig 24 presents an optical microscopy image of the wafer surface. Fig 25 shows the corresponding scanning electron microscopy (SEM) image confirming the homogeneity and size distribution of the pyramids formed on the wafer surface. The electrical performance of the fabricated solar cells is presented in Fig 26. Fig 26(a) shows the screenshot of the Solar Cell Module Tester (SCMT), while Fig 26(b) presents the corresponding MATLAB plot of the current–voltage (I–V) and power–voltage (P–V) characteristics derived from the measured data. Additionally, the complete voltage, current, and power measurement data are provided in S4 Appendix.

**Fig 13:**
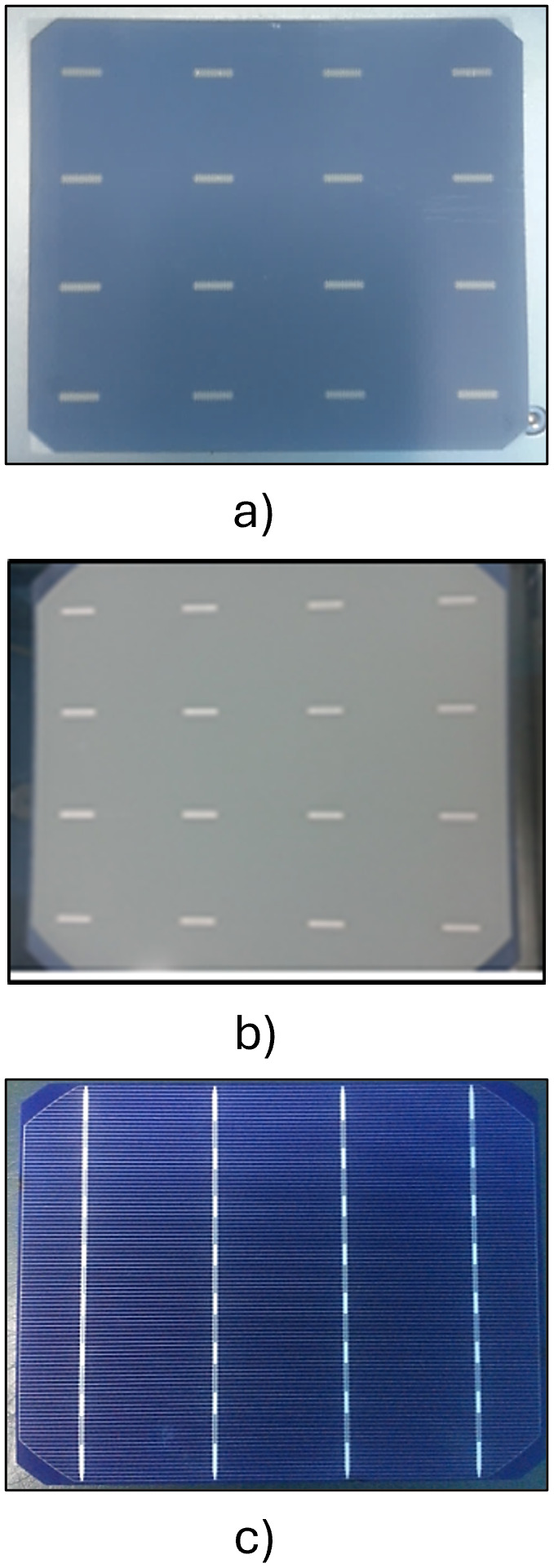
Wafer Appearance of each screen printer in the metallization sequence.

**Fig 14:**
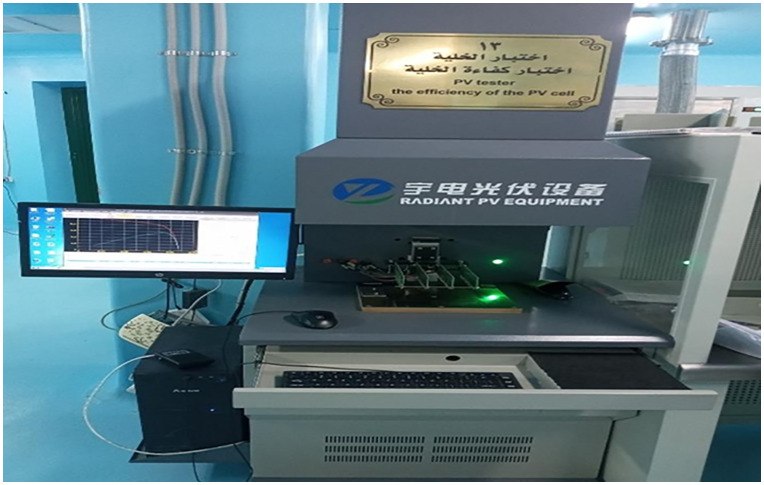
I–V tester for solar cell characterization.

**Fig 15:**
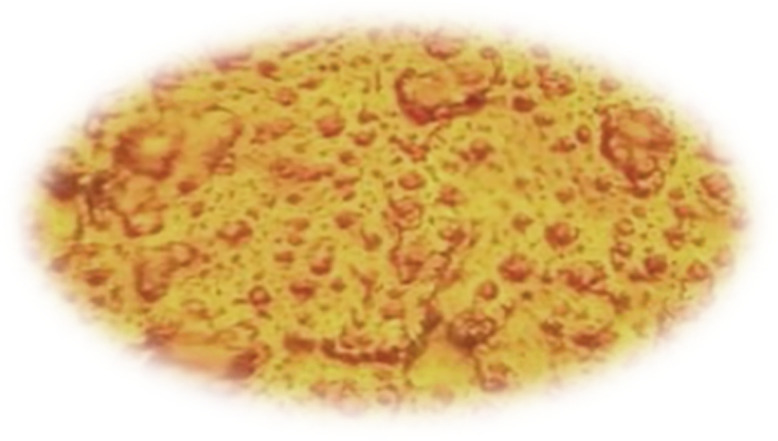
Texturing process without any additive material.

**Fig 16:**
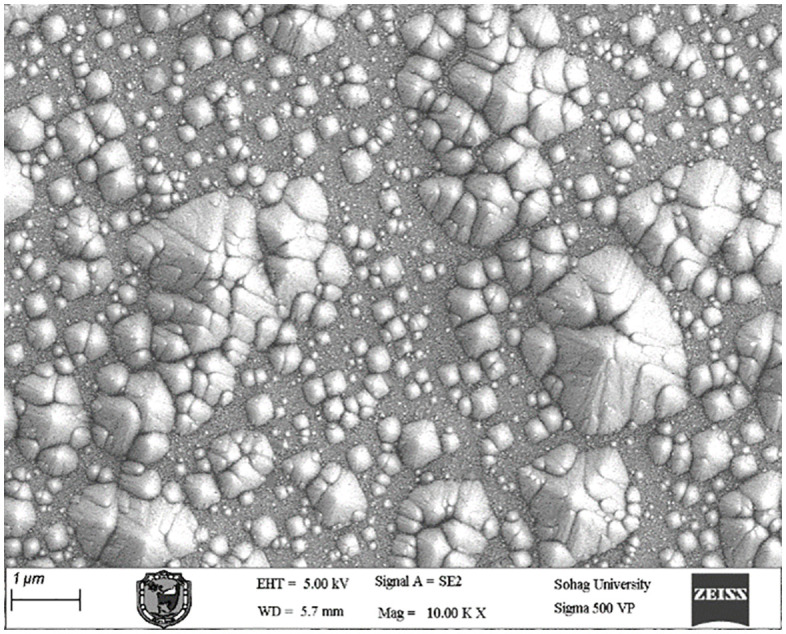
SEM image of silicon wafer surface for the case of texturing process without adding any active material.

**Fig 17:**
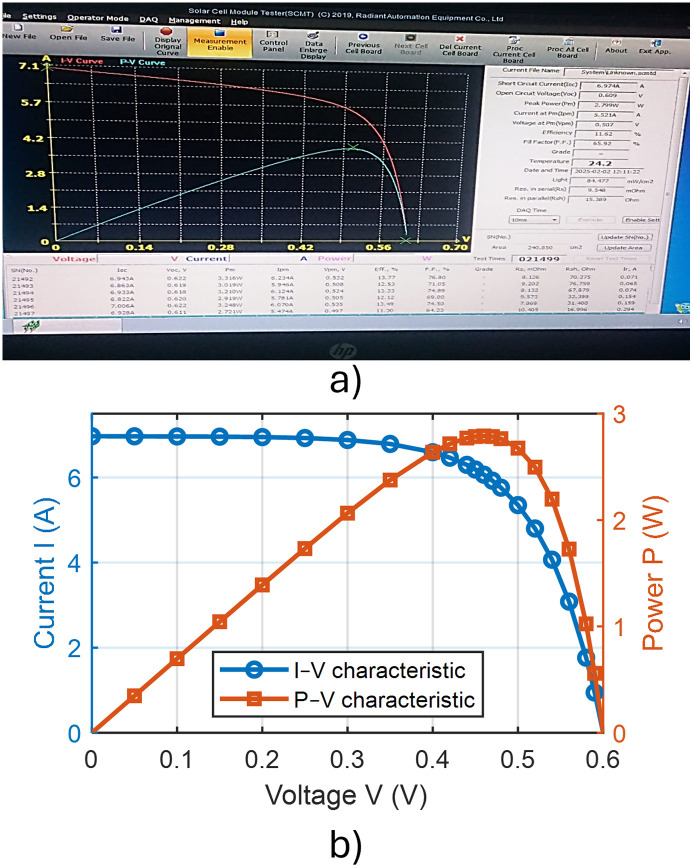
(a) Screenshot of the Solar Cell Module Tester (SCMT). (b) MATLAB plot of the measured I–V and P–V characteristics of the tested solar module for the case of texturing process without adding any active material.

**Fig 18:**
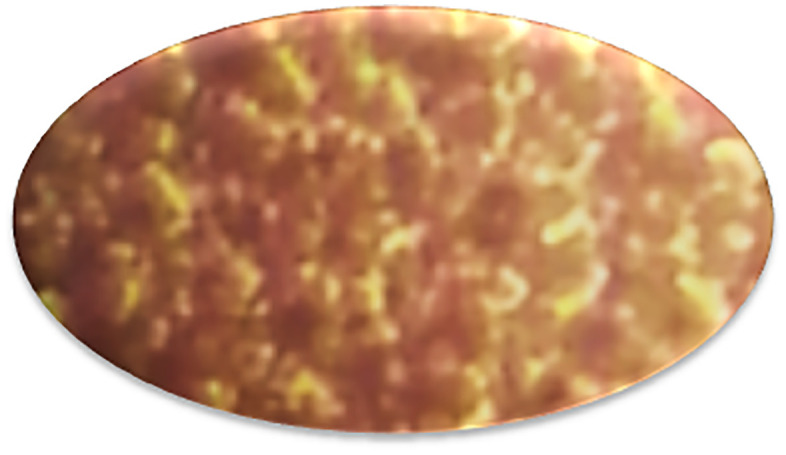
Texturing process with add 500 ml of the active material.

**Fig 19:**
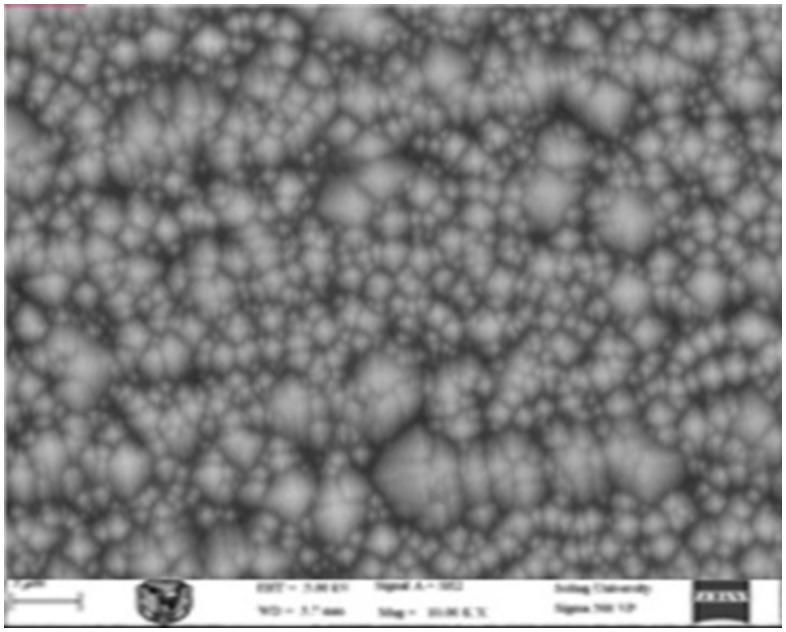
SEM image of silicon wafer surface for the case of texturing process with add 500 ml of active material (IPA).

**Fig 20:**
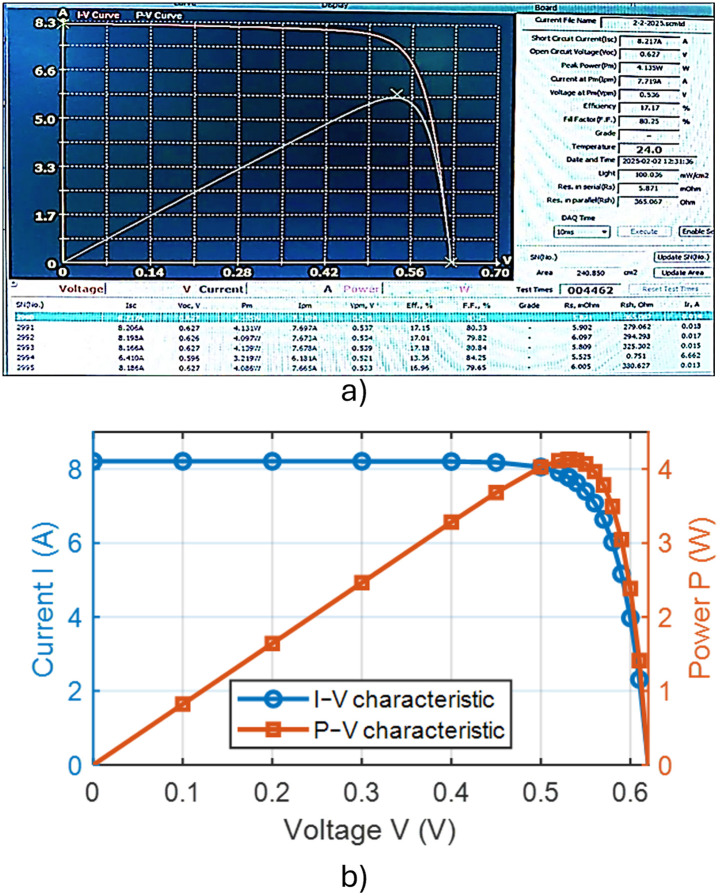
(a) Screenshot of the Solar Cell Module Tester (SCMT). (b) MATLAB plot of the measured I–V and P–V characteristics of the tested solar module for the case of texturing process with add 500 ml of active material (IPA).

**Fig 21:**
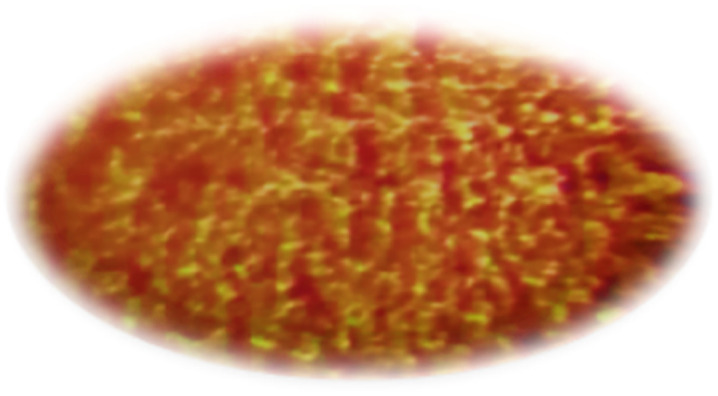
The texturing process adds 250 ml of active material (IPA), and 5 grams of potassium silicate.

**Fig 22:**
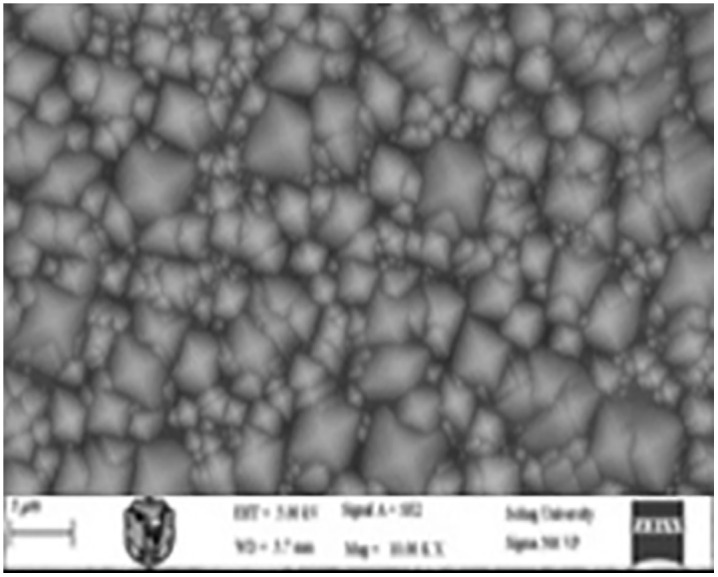
The SEM image of silicon wafer surface for the case of texturing process with 250 mL of Isopropyl Alcohol (IPA) and 5 g of Potassium Silicate.

**Fig 23:**
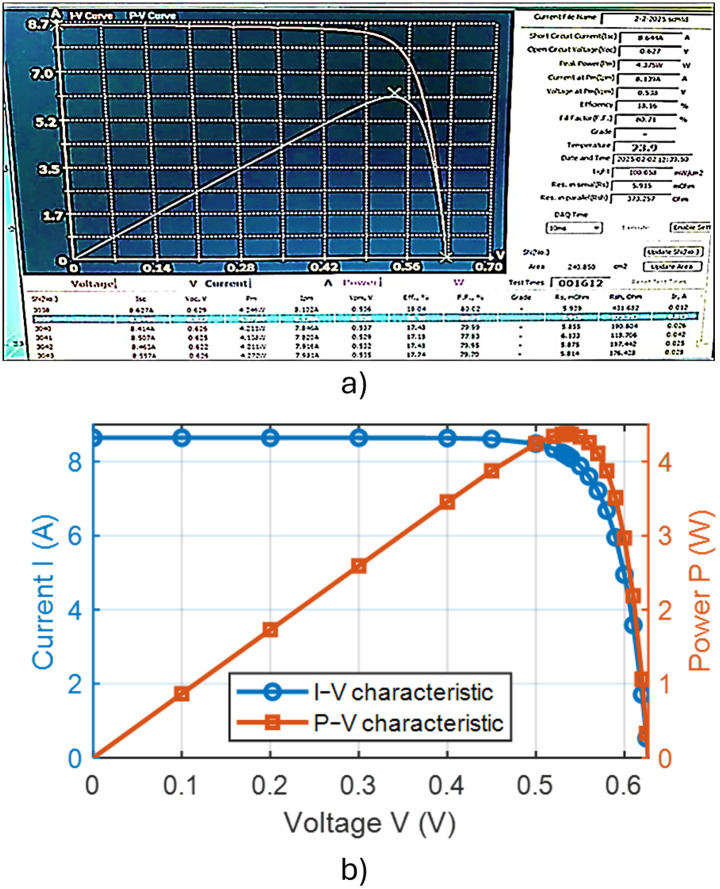
(a) Screenshot of the Solar Cell Module Tester (SCMT). (b) MATLAB plot of the measured I–V and P–V characteristics of the tested solar module for the case of texturing process with 250 mL of Isopropyl Alcohol (IPA) and 5 g of Potassium Silicate.

**Fig 24:**
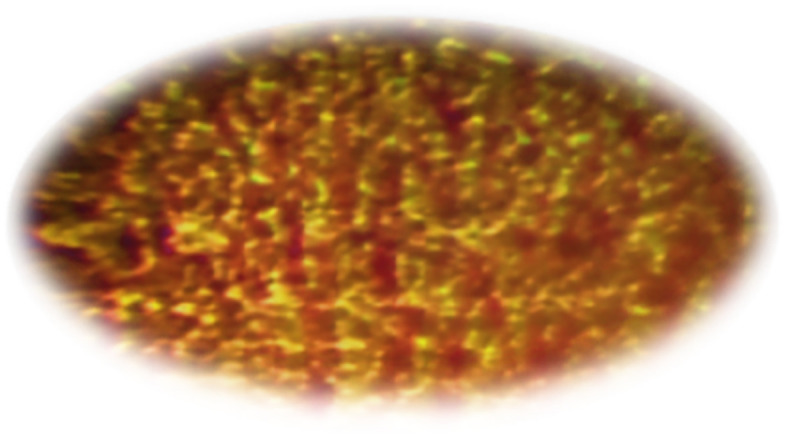
The texturing process with adds 250 ml of active material (IPA), and 10 grams of potassium silicate.

**Fig 25:**
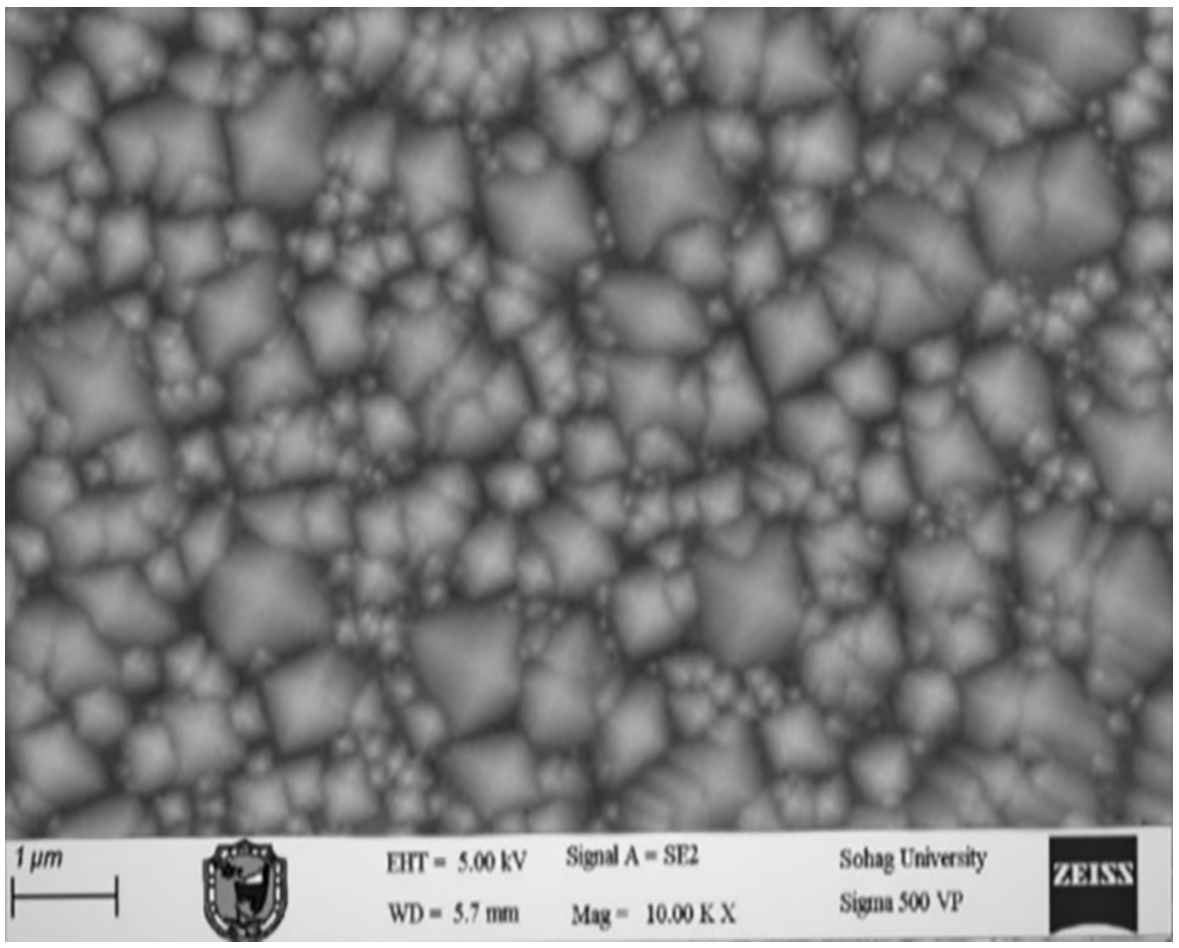
SEM image of silicon wafer surface for the case of texturing process with 250 mL of Isopropyl Alcohol (IPA) and 10 g of Potassium Silicate.

**Fig 26:**
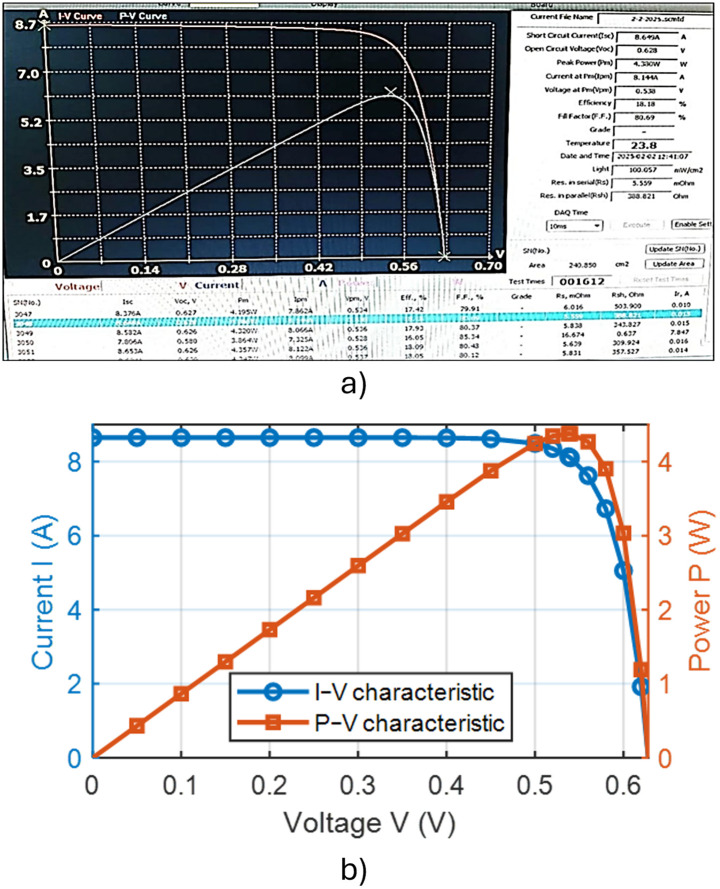
(a) Screenshot of the Solar Cell Module Tester (SCMT). (b) MATLAB plot of the measured I–V and P–V characteristics of the tested solar module for the case of texturing process with 250 mL of Isopropyl Alcohol (IPA) and 10 g of Potassium Silicate.

### 3.5 Comprehensive comparison of solar cell fabrication cases

**Fig 27:**
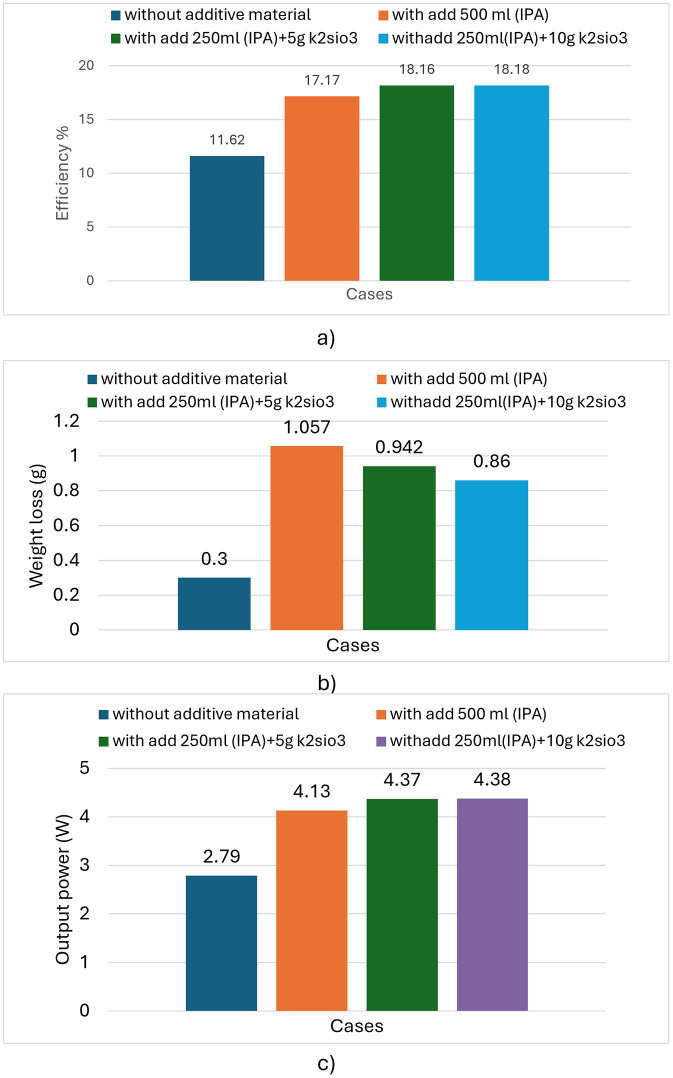
(a) Solar cell efficiency under different processing conditions; (b) Surface corrosion percentage of silicon wafers corresponding to each condition; (c) Output power for different processing conditions.

Fig 27a presents the measured efficiency of the fabricated solar cells for each experimental condition, highlighting the effect of process variations on energy conversion performance. Fig 26b shows the corresponding surface corrosion percentage observed on the silicon wafers in each case, reflecting the impact of chemical treatments on wafer integrity. Fig 27c illustrates the power output for each condition, with the maximum power achieved being 4.38 W.

The electrical and physical parameters of solar cells under four different processing conditions are compared in Table 3, including series resistance, shunt resistance, fill factor, efficiency, power output, and sheet resistance. The results demonstrate that in the absence of any active additive, the cells exhibit the lowest performance, with a fill factor (FF) of 65.92%, an efficiency (η) of 11.62%, and a maximum power (Pmax) of 2.79 W. The high series resistance (Rs = 9.54 mΩ) and low shunt resistance (Rsh = 15.3 Ω) indicate poor current collection and the presence of leakage paths. Sheet resistance was also highest under this condition, measuring 82.1 Ω/sq.

**Table 3:** Electrical and physical performance of solar cells under different chemical treatments.

Rsh (Ω)	Rs (mΩ)	FF (%)	Eff (%)	Pm (W)	Voc (V)	Isc (A)	Sheet resistance (Ω/sq)	Condition
15.3	9.54	65.92	11.62	2.79	0.600	6.97	82.10	Without active material
365	5.87	80.25	17.17	4.13	0.620	8.21	61.99	500 mL IPA
373	5.91	80.70	18.16	4.37	0.627	8.644	44.1	250 mL IPA + 5 g K₂SiO₃
388	5.55	80.70	18.18	4.38	0.628	8.649	44.92	250 mL IPA + 10 g K₂SiO₃

Upon addition of 500 mL of IPA, performance improved substantially. FF increased to 80.25% and efficiency reached 17.17%. Series resistance decreased to 5.87 mΩ, while shunt resistance rose sharply to 365 Ω, indicating improved junction quality and reduced leakage. Sheet resistance dropped to 61.99 Ω/sq, reflecting better surface conductivity. When the IPA volume was reduced to 250 mL and supplemented with 5 g of K₂SiO₃, efficiency increased further to 18.16%, with Pmax reaching 4.37 W and FF peaking at 80.7%. Series resistance was measured at 5.91 mΩ under this condition.

The highest performance was observed with the addition of 250 mL IPA and 10 g K₂SiO₃. Efficiency reached 18.18%, Pmax attained 4.38 W, and FF remained at 80.7%. Series resistance was further reduced to 5.55 mΩ, while sheet resistance decreased significantly to 44.92 Ω/sq. The observed sheet resistance reduction from 82.1 Ω/sq (no additive) to 44.92 Ω/sq (with 10 g K₂SiO₃) results from enhanced diffusion uniformity on more homogeneous pyramid surfaces. K₂SiO₃ promotes consistent pyramid formation, creating a more uniform substrate for phosphorus diffusion. This improved surface morphology enables more controlled emitter formation, contributing to the enhanced cell efficiency observed with K₂SiO₃ addition. Basu et al. [28] achieved efficiencies exceeding 18% by incorporating potassium silicate. The present study confirms and builds upon these findings: adding 5 g K₂SiO₃ with reduced IPA (250 mL) yielded a maximum efficiency of 18.16%, approaching the > 18% threshold reported by Basu et al. [28], while 10 g K₂SiO₃ produced 18.18%.

### 3.6 Statistical analysis

To investigate the impact of IPA and K₂SiO₃ additives on solar cell performance, four experimental groups were prepared: Sample A (without active material), Sample B (500 mL IPA only), Sample C (250 mL IPA + 5 g K₂SiO₃), and Sample D (250 mL IPA + 10 g K₂SiO₃). For each group, four independent measurements (n = 4) were conducted to ensure repeatability. The complete efficiency dataset is presented in Table 4, with corresponding descriptive statistics summarized in Table 5.

**Table 4:** Individual efficiency measurements (%) for all experimental conditions.

Efficiency of sample (A)	Efficiency of sample (B)	Efficiency of sample (C)	Efficiency of sample (D)
Without adding any active material	Adding 500 ml of (IPA)	Adding 250 ml of (IPA), and 5 g K₂SiO₃	Adding 250 ml of (IPA), and 10 g K₂SiO₃
A1 = 11.09	B1 = 16.91	C1 = 18.16	D1 = 18.17
A2 = 11.36	B2 = 17.17	C2 = 17.99	D2 = 18.09
A3 = 11.62	B3 = 16.89	C3 = 17.62	D3 = 18.18
A4 = 11.91	B4 = 17.28	C4 = 17.88	D4 = 18.17

**Table 5:** Statistical analysis of efficiency for different sample configurations.

Sample	Experimental Condition	Mean Efficiency	Standard Deviation	Minimum	Maximum	Range
A	Without adding any active material	11.50	0.35	11.09	11.91	0.82
B	Adding 500 ml IPA	17.06	0.19	16.89	17.28	0.39
C	Adding 250 ml IPA + 5 g K₂SiO₃	17.91	0.22	17.62	18.16	0.54
D	Adding 250 ml IPA + 10 g K₂SiO₃	18.15	0.04	18.09	18.18	0.09

The descriptive statistics reveal a clear trend of improving performance with additive incorporation. Sample A, prepared without any active material, exhibits the lowest mean efficiency (11.50%) and the highest variability (SD = 0.35%, range = 0.82%), indicating limited performance and reduced process stability. The introduction of IPA alone (Sample B) produces a substantial efficiency increase to 17.06%, representing an absolute improvement of 5.56% (approximately 48% relative increase) compared to Sample A. The reduced variability (SD = 0.19%) confirms IPA’s role in both enhancing efficiency and stabilizing the texturing process. Further gains are achieved with potassium silicate addition. Sample C (5 g K₂SiO₃) attains a mean efficiency of 17.91%—an absolute increase of 0.85% relative to Sample B—while maintaining low variability (SD = 0.22%). Sample D (10 g K₂SiO₃) delivers the highest mean efficiency of 18.15%, accompanied by the lowest standard deviation (0.04%) and narrowest range (0.09%) among all conditions. These metrics demonstrate superior performance. The following sources of potential variability were identified for Sample D, which demonstrated the lowest standard deviation (0.04%):

- Although all wafers were sourced from the same batch with nominally identical specifications (Table 1) to analysis wafer-to-wafer variability, inherent variations in crystallographic defects, saw damage, and surface contamination can influence texturing outcomes. The low within-group variance suggests that such wafer-to-wafer differences were minimal under the controlled laboratory conditions, but larger-scale production environments may introduce greater heterogeneity.
- The texturing process was conducted at 85 °C, as optimized by the Chinese training team. Temperature gradients within or between tanks could affect etch rates.
- All four experimental conditions were processed sequentially within the same laboratory session, which minimizes batch-to-batch variability.
- The efficiency measurements were obtained using an I–V tester under standard conditions (25 °C, AM 1.5, 1000 W/m²). While the instrument is calibrated, systematic uncertainties (e.g., lamp uniformity, temperature control during testing) could contribute to measurement variability.

#### 3.6.1 Analysis of variance (ANOVA).

To determine whether the observed differences among the four experimental groups were statistically significant, a one-way analysis of variance (ANOVA) was performed. The results are presented in Table 6**.**

**Table 6:** One-way ANOVA results for solar cell efficiency across processing conditions.

Source of Variation	SS	df	MS	F	P-value	F crit
Between Groups	118.4719	3	39.49062	739.1499	p < 0.001	3.490295
Within Groups	0.641125	12	0.053427			
Total	119.113	15				

The ANOVA results reveal a calculated F-value of 739.15, which substantially exceeds the critical value (F₀.₀₅ (3,12) ≈ 3.49) at the 0.05 significance level. The corresponding p-value (p < 0.001) indicates that the differences among group means are highly statistically significant, leading to rejection of the null hypothesis that all group means are equal. This confirms that the variations in efficiency are not attributable to random chance but are strongly influenced by the composition of the texturing solution. The results are consistent with the descriptive statistics. Sample A, which contains no active material, shows the lowest mean efficiency (~11.50), while Sample D, containing 250 ml of IPA and 10 g of K₂SiO₃, achieves the highest mean efficiency (~18.15). The very low within-group variance further highlights that these differences are statistically robust and visually clear.

**Fig 28:**
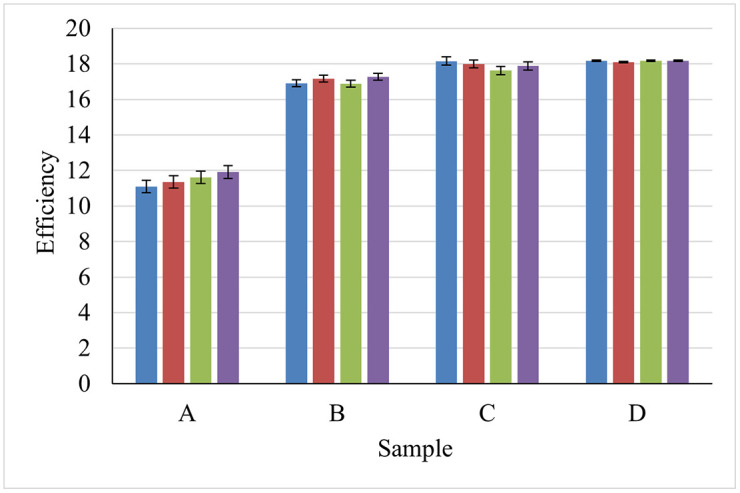
Mean efficiency values for solar cells fabricated under different additive conditions.

The presented statistical results confirm that both IPA and K₂SiO₃ have a significant and measurable effect on efficiency, with the addition of potassium silicate contributing to improved performance. Despite sources of potential variability were identified for Sample D, which demonstrated the lowest standard deviation (0.04%), the ANOVA results demonstrate that the within-group variance (MS_within = 0.0534) is exceptionally low, indicating that the combination of careful experimental control and consistent wafer quality successfully minimized extraneous variability. The statistical significance of the between-group differences (p < 0.001) confirms that the observed efficiency improvements are robust relative to the residual variability in the measurements. Fig 28 shows the efficiency that can illustrate the relationship between the addition of the active ingredient and the resulting solar cell efficiency in each case to visualize the differences between sample groups. To provide a complete representation of data variability, all figures presenting mean efficiency values now include error bars representing the standard deviation (SD) of the four independent measurements (n = 4 per group). While the sample size is modest, the one-way ANOVA revealed highly significant differences among groups (F(3,12) = 739.15, p < 0.001). The large F-value and small within-group variance (MSwithin = 0.053) indicate that the effect sizes of the additive treatments are substantial, allowing for the detection of significant differences even with this limited number of replicates. Nevertheless, future studies with larger sample sizes would be valuable to further enhance the statistical power and generalizability of these findings.

#### 3.6.2 Tukey HSD post-hoc analysis.

Following the one-way ANOVA, which revealed significant differences among the four experimental groups (F(3,12) = 739.15, p < 0.001), a Tukey Honestly Significant Difference (HSD) post-hoc test was conducted to identify specific pairwise differences. The analysis employed a family-wise error rate of α = 0.05, with a critical studentized range value of q(4,12) = 4.20. For this balanced design (n = 4 per group), the standard error for mean differences was calculated as:


$$\begin{array}{c} SE=\sqrt{\frac{{MS}_{within}}{n}}=\sqrt{\frac{\text{0}.\text{0}\text{5}\text{3}\text{4}\text{2}\text{7}}{\text{4}}}=\text{0}.\text{1}\text{1}\text{5}\text{6} \end{array}$$

The minimum significant difference (HSD) was therefore:


$$\begin{array}{c} HSD=q_{critical}\times SE=\text{4}.\text{2}\text{0}\times \text{0}.\text{1}\text{1}\text{5}\text{6}=\text{0}.\text{4}\text{8}\text{5}\text{5} \end{array}$$

Any absolute difference between two group means exceeding 0.4855 is statistically significant at the α = 0.05 level. The complete pairwise comparison results are presented in Table 7.

**Table 7:** Tukey HSD post-hoc analysis for pairwise comparisons of solar cell efficiency across processing conditions.

Significant (α = 0.05)	p-value	q-value	SE	Difference (I-J)	Comparison (I-J)
YES	p < 0.001	48.10	0.1156	5.56	B vs. A
YES	p < 0.001	55.45	0.1156	6.41	C vs. A
YES	p < 0.001	57.52	0.1156	6.65	D vs. A
YES	p < 0.001	7.35	0.1156	0.85	C vs. B
YES	p < 0.001	9.43	0.1156	1.09	D vs. B
NO	0.187	2.08	0.1156	0.24	D vs. C

The post-hoc results confirm that:

- All additive-containing groups (B, C, and D) exhibit significantly higher efficiencies than the additive-free control (A) (p < 0.001 for all comparisons). This confirms that the addition of IPA and/or K₂SiO₃ dramatically improves solar cell performance.
- Both potassium silicate-treated groups (C and D) significantly outperform the IPA-only group (B) (p < 0.001). This demonstrates that silicate addition provides a statistically meaningful enhancement beyond conventional IPA processing, with mean improvements of 0.85% and 1.09% for 5 g and 10 g concentrations, respectively.

### 3.7 Recommendations

This work demonstrates the successful fabrication of mono-Si solar cells, achieving an efficiency of 18.18% at the Egyptian-Chinese Laboratory in Sohag. This represents a significant milestone as the first reported instance of such fabrication in Egypt. A systematic analysis was conducted to correlate each step of the fabrication process with the final cell performance. The findings underscore the critical role of the texturing process in determining overall efficiency. By establishing a foundational manufacturing capability, this study paves the way for further efficiency improvements and supports the expansion of local photovoltaic manufacturing. Future research will focus on optimizing the texturing process through the integration of novel materials and on advancing the subsequent stages of the cell fabrication sequence within the laboratory.

## 4 Conclusion

This study demonstrates that the concentration and composition of active additives critically influence the texturing process for monocrystalline silicon solar cells, with direct consequences for surface morphology and photovoltaic performance. When isopropyl alcohol (IPA) was omitted from the etching solution, the texturing reaction exhibited negligible activity, resulting in insufficient surface corrosion and poor light-trapping capability; corresponding solar cells achieved a markedly low efficiency of 11.62%, confirming IPA’s essential role in initiating pyramid nucleation. The incorporation of potassium silicate (K₂SiO₃) as a co-additive resulted in a 50% reduction in IPA consumption and improved texturing quality. Specifically, the modified solution containing 250 mL of IPA and either 5 g or 10 g of K₂SiO₃ yielded highly uniform pyramidal structures with enhanced geometric regularity, in contrast to the morphological heterogeneity exhibited by the conventional formulation containing 500 mL of IPA. This improved homogeneity directly correlates with enhanced light absorption and carrier collection, yielding a peak efficiency of 18.18%—representing an absolute increase of 4.56% over the IPA-free baseline and a relative improvement of approximately 1% over the conventional IPA-only process (17.17%). Moreover, the addition of small quantities of liquid potassium silicate to the conventional KOH/IPA alkaline texturing process offers a multifunctional approach to optimizing both economic and performance metrics in solar cell fabrication. By reducing IPA consumption while improving texturing reproducibility and uniformity, this method aligns with industrial priorities for scalable, cost-effective photovoltaic manufacturing. Future studies should investigate the kinetic mechanisms governing K₂SiO₃ interaction with silicon surfaces and assess its applicability in large-scale production environments.

## Supporting information

S1 Appendix Data of Fig 16: Voltage, current, power data of Fig 16. (XLSX)

S2 Appendix Data of Fig 19: Voltage, current, power data of Fig 19. (XLS)

S3 Appendix Data of Fig 22: Voltage, current, power data of Fig 22. (XLSX)

S4 Appendix Data of Fig 25: Voltage, current, power data of Fig 25. (XLSX)

## References

[1] Chen J, Su F, Jain V, Salman A, Tabash MI, Haddad AM, et al. Does Renewable Energy Matter to Achieve Sustainable Development Goals? The Impact of Renewable Energy Strategies on Sustainable Economic Growth. Front Energy Res. 2022;10. doi: 10.3389/fenrg.2022.829252

[2] Koloba HA. Purchase intention towards environmentally friendly products among consumers in South Africa: Applying the theory of planned behaviour. International Journal of Business and Management Studies, 2020;12(1):34–49.

[3] Sen A. Promising DSSCs Involving Organic D–π–A and Similar Structures for n- and p-type Semiconductors—A Theoretical Approach. Challenges and Advances in Computational Chemistry and Physics. Springer International Publishing. 2021. 127–65. 10.1007/978-3-030-69445-6_6

[4] Strielkowski W, Civín L, Tarkhanova E, Tvaronavičienė M, Petrenko Y. Renewable Energy in the Sustainable Development of Electrical Power Sector: A Review. Energies. 2021;14(24):8240. doi: 10.3390/en14248240

[5] Kılıç U, Kekezoğlu B. A review of solar photovoltaic incentives and Policy: Selected countries and Turkey. Ain Shams Engineering Journal. 2022;13(5):101669. doi: 10.1016/j.asej.2021.101669

[6] Chander S, Purohit A, Sharma A, Arvind, Nehra SP, Dhaka MS. A study on photovoltaic parameters of mono-crystalline silicon solar cell with cell temperature. Energy Reports. 2015;1:104–9. doi: 10.1016/j.egyr.2015.03.004

[7] Chang X, Wu Z, Wang J, Zhang X, Zhou M, Yu T, et al. The coupling effect of carbon emission trading and tradable green certificates under electricity marketization in China. Renewable and Sustainable Energy Reviews. 2023;187:113750. doi: 10.1016/j.rser.2023.113750

[8] Shukla PR. Climate Change 2022: Mitigation of Climate Change. Cambridge, UK and New York, NY, USA: Cambridge University Press. 2022. 10.1017/9781009157926

[9] IRENA. Renewable Capacity Statistics 2025. Abu Dhabi: International Renewable Energy Agency. 2025.

[10] Raval MC, Joshi AP, Saseendran SS, Suckow S, Saravanan S, Solanki CS, et al. Study of Nickel Silicide Formation and Associated Fill-Factor Loss Analysis for Silicon Solar Cells With Plated Ni-Cu Based Metallization. IEEE J Photovoltaics. 2015;5(6):1554–62. doi: 10.1109/jphotov.2015.2463741

[11] Murukeshan K, et al. POCl₃ diffusion process optimization for the formation of emitters in crystalline silicon solar cells. In: 2014 IEEE 40th Photovoltaic Specialist Conference (PVSC), 2014. 3011–3.

[12] Schultz O, Glunz SW, Riepe S, Willeke GP. High-efficiency solar cells on phosphorus gettered multicrystalline silicon substrates. Prog Photovolt: Res Appl. 2006;14(8):711–9. doi: 10.1002/pip.736

[13] Koynov S, Brandt MS, Stutzmann M. Black nonreflecting silicon surfaces for solar cells. Applied Physics Letters. 2006;88(20). doi: 10.1063/1.2204573

[14] Liu Y, Das A, Lin Z, Cooper IB, Rohatgi A, Wong CP. Hierarchical robust textured structures for large scale self-cleaning black silicon solar cells. Nano Energy. 2014;3:127–33. doi: 10.1016/j.nanoen.2013.11.002

[15] Sievert W, Zimmermann KU, Hartmann B, Klimm C, Jacob K, Angermann H. Surface Texturization and Interface Passivation of Mono-Crystalline Silicon Substrates by Wet Chemical Treatments. SSP. 2009;145–146:223–6. doi: 10.4028/www.scientific.net/ssp.145-146.223

[16] Khalil IM, Mohamed AA. Enhancing solar panel performance using a nano-porous silica anti-reflective layer fabricated via sol-gel process. Journal of Advanced Engineering Trends. 2023;42(2):101–12.

[17] Hamdy MG, Mohamed RB, Maher H. Analytical and computational model for predicting the thermal performance of PV cells. J Adv Eng Trends. 2025;44(1):291–7. doi: 10.21608/jaet.2024.292103.1288

[18] Elsheikh A, El-Said EM, Abd Elaziz M. Performance enhancement of photovoltaic panels using integrated phase change materials: An experimental study in Minia, Egypt. Journal of Advanced Engineering Trends. 2022;41(1):55–67.

[19] Chen N, Liu Y, Shao Y, Hu X, Zhang X, Liu R, et al. Review of the latest industrial progress in screen printing. Solar Energy Materials and Solar Cells. 2025;290:113734. doi: 10.1016/j.solmat.2025.113734

[20] Saseendran SS, Saravanan S, Raval MC, Kottantharayil A. Impact of interstitial oxygen trapped in silicon during plasma growth of silicon oxy-nitride films for silicon solar cell passivation. Journal of Applied Physics. 2016;119(9). doi: 10.1063/1.4943177

[21] Park H, Kwon S, Lee JS, Lim HJ, Yoon S, Kim D. Improvement on surface texturing of single crystalline silicon for solar cells by saw-damage etching using an acidic solution. Solar Energy Materials and Solar Cells. 2009;93(10):1773–8. doi: 10.1016/j.solmat.2009.06.012

[22] Papet P, Nichiporuk O, Kaminski A, Rozier Y, Kraiem J, Lelievre J-F, et al. Pyramidal texturing of silicon solar cell with TMAH chemical anisotropic etching. Solar Energy Materials and Solar Cells. 2006;90(15):2319–28. doi: 10.1016/j.solmat.2006.03.005

[23] Chen K, Liu Y, Wang X, Zhang L, Su X. Novel texturing process for diamond-wire-sawn single-crystalline silicon solar cell. Solar Energy Materials and Solar Cells. 2015;133:148–55. doi: 10.1016/j.solmat.2014.11.016

[24] Acker J, Koschwitz T, Meinel B, Heinemann R, Blocks C. HF/HNO3 Etching of the Saw Damage. Energy Procedia. 2013;38:223–33. doi: 10.1016/j.egypro.2013.07.271

[25] Campbell P, Green MA. Light trapping properties of pyramidally textured surfaces. Journal of Applied Physics. 1987;62(1):243–9. doi: 10.1063/1.339189

[26] Rodriguez J. Random pyramidal texture modelling. Solar Energy Materials and Solar Cells. 1997;45(3):241–53. doi: 10.1016/s0927-0248(96)00040-2

[27] Vazsonyi E, De Clercq K, Einhaus R, Van Kerschaver E, Said K, Poortmans J, et al. Improved anisotropic etching process for industrial texturing of silicon solar cells. Solar Energy Materials and Solar Cells. 1999;57(2):179–88. doi: 10.1016/s0927-0248(98)00180-9

[28] Basu PK, Sarangi D, Shetty KD, Boreland MB. Liquid silicate additive for alkaline texturing of mono-Si wafers to improve process bath lifetime and reduce IPA consumption. Solar Energy Materials and Solar Cells. 2013;113:37–43. doi: 10.1016/j.solmat.2013.01.037

[29] Birmann K, Zimmer M, Rentsch J. Fast alkaline etching of monocrystalline wafers in KOH/CHX. In: Proceedings of the 23rd European Photovoltaic Solar Energy Conference and Exhibition, Valencia, Spain, 2008. 1608–11.

[30] Mayer K. Aromatic compounds as substitutes for 2-propanol as texturing additive in aqueous alkaline solutions. In: Valencia, Spain, 2010.

[31] Ximello N, et al. Up to 20% efficient solar cells on monocrystalline silicon wafers by using a KOH-high boiling alcohol (HBA) texturing solution. Bibliothek der Universität Konstanz. 2011.

[32] Lee J, Lakshminarayan N, Dhungel SK, Kim K, Yi J. Optimization of fabrication process of high-efficiency and low-cost crystalline silicon solar cell for industrial applications. Solar Energy Materials and Solar Cells. 2009;93(2):256–61. doi: 10.1016/j.solmat.2008.10.013

[33] Basu PK. Alkaline pyramidal texturing processes for industrial monocrystalline silicon wafer solar cells. SMC Bulletin. 2017;8(2).

[34] Han S, Chu M, Pham DP, Dhungel SK, Yi J. Comparison of different approaches to texturing monocrystalline silicon wafers for solar cell applications. Surface Science. 2024;748:122540. doi: 10.1016/j.susc.2024.122540

